# Dual Role of SIRT1 in Autophagy and Lipid Metabolism Regulation in Osteoarthritic Chondrocytes

**DOI:** 10.3390/medicina57111203

**Published:** 2021-11-04

**Authors:** Aliki-Alexandra Papageorgiou, Andreas Goutas, Varvara Trachana, Aspasia Tsezou

**Affiliations:** 1 Laboratory of Cytogenetics and Molecular Genetics, Faculty of Medicine, University of Thessaly, Biopolis, 41500 Larissa, Greece; alipapageorgiou@med.uth.gr; 2 Department of Biology, Faculty of Medicine, University of Thessaly, Biopolis, 41500 Larissa, Greece; agoutas@uth.gr (A.G.); vtrachana@med.uth.gr (V.T.)

**Keywords:** osteoarthritis, sirtuin 1, lipid metabolism, autophagy

## Abstract

*Background and Objectives*: Osteoarthritis (OA) is one of the most common and highly prevalent types of arthritis, also considered a multiphenotypic disease with a strong metabolic component. Ageing is the primary risk factor for OA, while the age-related decline in autophagic activity affects cell function and chondrocyte homeostasis. The aim of this study was to investigate the role of sirtuin 1 (SIRT1) in autophagy dysregulation and lipid metabolism in human OA chondrocytes. *Materials and Methods*: OA chondrocytes were treated with Resveratrol, Hydroxycloroquine (HCQ) or 3-Methyladenine (3-MA) and HCQ or 3-MA followed by siRNA against SIRT1 (siSIRT1). Then, SIRT1, AcNF-κBp65, LOX-1 and autophagy-related proteins ATG5, ATG13, PI3K class III, Beclin-1, LC3 and ULK protein levels were evaluated using Western blot. Normal articular chondrocytes were treated under serum starvation and/or siSIRT1, and the protein expression levels of the above autophagy-related proteins were evaluated. The staining patterns of LC3/p62 and LOX-1 were analyzed microscopically by immunofluorescence. SIRT1/LC3 complex formation was analyzed by immunoprecipitation. *Results*: SIRT1 and LOX-1 protein expression were negatively correlated in OA chondrocytes. SIRT1 regulated LOX-1 expression via NF-κΒ deacetylation, while treatment with Resveratrol enhanced SIRT1 enzymatic activity, resulting in LOX-1 downregulation and autophagy induction. In OA chondrocytes, SIRT1 was recognized as an autophagy substrate, formed a complex with LC3 and was consequently subjected to cytoplasmic autophagosome-lysosome degradation. Moreover, siSIRT1-treated normal chondrocytes showed decreased autophagic activity, while double-treated (siSIRT1 and serum starvation) cells showed no induction of autophagy. *Conclusions*: Our results suggest that SIRT1 regulates lipid homeostasis through LOX-1 expression regulation. Additionally, we indicate that the necessity of SIRT1 for autophagy induction in normal chondrocytes, together with its selective autophagic degradation in OA chondrocytes, could contribute to autophagy dysregulation in OA. We, therefore, suggest a novel regulatory scheme that functionally connects lipid metabolism and autophagy in late-stage OA.

## 1. Introduction

Osteoarthritis (OA) is whole-joint disorder characterized by the loss of articular cartilage of synovial joints, synovial inflammation, subchondral bone remodeling and ligament fibrosis [1,2]. It is the most common type of arthritis worldwide [3], with no effective treatment so far [4]. OA is a complex disease, with several environmental and systemic factors being major contributors to its onset and development. Among them, ageing is one of the primary risk factors for OA [5], while it also has a systemic metabolic component [6,7]. Central obesity, frequently associated with metabolic syndrome (MetS) [8], increases OA prevalence [9], as the majority of the elderly and obese population suffers from knee OA [10].

Many chronic and metabolic diseases, including OA, have been associated with autophagy deregulation [11,12]. Autophagy is a lysosomal degradative pathway responsible for the recycling of macromolecules and cytoplasmic material for biosynthetic or energy-related purposes [13,14]. Autophagy is mainly a cytoprotective mechanism, as it is responsible for the removal of dysfunctional macromolecules and organelles, which can arise through various kinds of stresses [15,16]. It can be triggered by nutrient deprivation (serum starvation) [17] or cytotoxic insults and damaged proteins and organelles. The process is strictly regulated mainly by the action of two distinct complexes, ULK1 and PI3K-CIII. The targets of these two kinases (ULK1 and PI3K-CIII) include several regulatory factors, such as Beclin-1 and other autophagy-related (ATG) proteins [18], to promote the early steps of phagophore biogenesis. After the first two steps (initiation, nucleation), the third step is elongation, and in order for this to take place, two ubiquitin-like systems are necessary: the ATG5 complex and the conjugation of microtubule-associated protein 1 light chain 3 (LC3). Several other proteins contribute to maturation process of the phagophore to autophagosome that is then destined to fuse to a lysosome, leading to cargo degradation and the recycling of nutrients and metabolites [19,20].

In OA, autophagy decreases with age, as shown by the reduced expression levels of ULK1, Beclin-1 and LC3 in OA articular cartilage, resulting in the accumulation of dysfunctional proteins and organelles in OA chondrocytes [21]. However, it has been reported to have a dual role in OA pathogenesis [22] as it can reduce cell death at early disease stages, while at late-stage OA, excessive autophagy may cause cell death [11,23].

Furthermore, autophagy has been shown to be a critical regulator of lipid metabolism, suggesting a possible interplay between autophagy and lipid metabolism [24]. Lipophagy, the autophagic mechanism responsible for the lysosomal degradation of intracellular lipids, has been demonstrated to be crucial during nutrient starvation. Thus, when inactivated, lipids stored in lipid droplets (LDs) accumulate in adipocytes [25,26,27]. On the other hand, recent studies reported that excessive lipid uptake and ox-LDL accumulation in OA chondrocytes could facilitate tumor necrosis factor α (TNF α)-mediated chondrocyte death via an autophagy pathway, mainly through the action of lectin-like oxidized low-density lipoprotein receptor-1 (LOX-1) [28]. Essentially, when acetylated NF-κB binds to the LOX-1 promoter and induces LOX-1 transcription [29,30], SIRT1 targets NF-κB for deacetylation, reducing its binding affinity, disabling ox-LDL scavenger receptor LOX-1 transcription and resulting, thus, in reduced ox-LDL uptake [31,32].

SIRT1, a NAD+-dependent deacetylase, is a key regulator of a broad range of cellular processes that have been associated with OA [33], including the metabolic response and ageing [34,35,36,37]. SIRT1 expression exhibits an age-related decline, as it is highly expressed in younger adults and is decreased in elderly OA patients [38]. Decreased SIRT1 expression in OA chondrocytes has been associated with abnormal chondrocyte biology, increased catabolic activity and decreased chondrogenic markers, along with key autophagy markers and metabolic imbalance [39,40]. However, SIRT1’s involvement in OA chondrocyte lipid metabolism is poorly investigated. In the present study, we aim to investigate the implication of SIRT1 in autophagy induction and lipid metabolism in OA chondrocytes

## 2. Materials and Methods

### 2.1. Patients and Tissue Samples

Human articular cartilage specimens were obtained from the femoral condyles and tibial plateaus of 20 patients with primary OA (15F/5M; mean age 63.25 ± 7.1 years) undergoing knee replacement surgery at the Department of Orthopaedics of the University Hospital of Larissa. All osteoarthritic cartilage samples were taken from the main defective area with visible severe degeneration (advanced OA) and focal or diffuse exposure of subchondral bone, as well as from adjacent cartilage tissue with minimal lesions (early OA). Radiographs were obtained before surgery and the Kellgren-Lawrence (K/L) grading system was used for the assessment of OA severity. All patients had K/L score > 2. The assessment of the radiographs by two independent experts was blinded. Patients with rheumatoid arthritis or other autoimmune diseases, as well as chondrodysplasias, septic or post-traumatic OA, were excluded from the study. Normal articular cartilage samples were obtained from 10 individuals (7F/3M; mean age 51.4 ± 4.4 years) undergoing knee fracture repair surgery or amputation surgery, with no history of joint disease and no clinical manifestations compatible with OA. All samples were collected from the Department of Orthopaedics of the University Hospital of Larissa.

### 2.2. Primary Cultures of Normal and OA Human Articular Chondrocytes

Following dissection, articular cartilage tissues were subjected to sequential digestion with 1 mg/mL pronase and 1 mg/mL collagenase P (Roche Applied Science, Mannheim, Germany). Isolated chondrocytes from individual specimens of OA patients (hereafter OA) and healthy donors (hereafter Normal) were separately cultured with Dulbecco’s Modified Eagle’s Medium/Ham’s F-12 (DMEM/F-12) (Thermo Fisher Scientific, Waltham, MA, USA) supplemented with 10% FBS (FBS, GIBCO, Thermo Fisher Scientific, USA) and 1% antibiotics (penicillin–streptomycin, GIBCO, Thermo Fisher Scientific, USA) at 37 °C, in a humidified 5% CO_2_ atmosphere.

### 2.3. Treatment of Normal Chondrocytes with siRNA against SIRT1 under Serum Starvation Conditions

Normal articular chondrocytes were counted and seeded onto six-well plates at a density of 3 × 10^5^ cells/well. On the day of the experiment, the FBS-containing medium was removed, and cells were carefully washed twice with warm, sterile PBS. Chondrocytes were subsequently serum-deprived in FBS-free DMEM/F-12 culture medium, for 2 h. For transfection experiments, DMEM/F-12 was removed, cells were plated in 500 μL Opti-MEM (GIBCO, Thermo Fisher Scientific, USA) and 100 pmol of siRNA against SIRT1 (Thermo Fisher Scientific, USA) was added in normal chondrocytes for 48 h. Transfection was performed using Lipofectamine 2000 reagent (Thermo Fisher Scientific, USA) according to the manufacturer’s protocol.

### 2.4. Treatment of OA Chondrocytes with Resveratrol, Hydroxychloroquine, 3-Methyladenine and siRNA against SIRT1

OA articular chondrocytes were counted and seeded onto six-well plates at a density of 3 × 10^5^ cells/well. Resveratrol (Tocris Bioscience, Bio-Techne Corporation, Minneapolis, MN, USA) was prepared as a 100 mM solution in DMSO and was further diluted in cell culture medium to achieve the final working concentrations. Resveratrol (25 μΜ) was added to cells for 72 h, while DMSO was used as a control treatment. OA chondrocytes were, also, treated with Hydroxychloroquine (HCQ) for 2 h. HCQ was prepared as a 20 mM stock solution, and was further diluted in culture medium to achieve a 100 μΜ concentration. Moreover, as an alternative method to inhibit autophagy, OA chondrocytes were treated with a second commonly used autophagy inhibitor, 3-Methyladenine (3-MA), for 2 h. In this case, 3-MA was prepared as a 25 mM stock solution, and was further diluted in culture medium to achieve a 10 μΜ concentration. Following treatment with Resveratrol, HCQ and 3-MA, cells were harvested for protein extraction. Next, siRNA transfection was performed as previously described, followed by HCQ or 3-MA treatment, 2 h prior to chondrocyte harvesting.

### 2.5. Western Blot Analysis

Chondrocytes were lysed in RIPA buffer (10 mM Tris (pH 7.5), 150 mM NaCl, 1% Triton X-100, 1% Sodium Deoxycholated, 0.1% SDS, 1 mM EDTA) supplemented with a protease inhibitor cocktail (Roche Applied Science, Mannheim, Germany). Protein concentrations were quantified using the Bio-Rad Bradford Protein assay (Bio-Rad Protein Assay, BioRad, Hercules, CA, USA) with bovine serum albumin as a standard. Samples (20–50 μg total protein) were analyzed by 8–12% SDS–PAGE according to standard procedures and transferred to PVDF membranes (Millipore, Billerica, MA, USA). Membranes were blocked with 5% *w/v* non-fat dry milk dissolved in PBS/0.1%Tween20. Membranes were incubated overnight with the primary antibody at the appropriate dilution each time, in blocking buffer at 4 °C. The antibodies used were anti-SIRT1 (1:1000, Cell Signaling Technology, Rabbit #2496), anti-LC3 (1:1000 dilution, Cell Signaling Technology, LC3A/B Rabbit #4108), p62 (1:1000 dilution, Cell Signaling Technology, SQSTM1/p62 Mouse #88588), Beclin-1 (1:1000 dilution, Cell Signaling Technology, Beclin-1 Rabbit #3495), PI3K-CIII (1:1000 dilution, Cell Signaling Technology, PI3 Kinase Class III Rabbit #4263), ATG5 (1:1000 dilution, Cell Signaling Technology, ATG5 Rabbit #12994), ATG13 (1:1000 dilution, Cell Signaling Technology, ATG13 Rabbit #13468), ULK1 (1:1000 dilution, Cell Signaling Technology, ULK1 Rabbit #8054), anti-Acetyl-NF-κB p65 (1:500 dilution, Cell Signaling Technology, Rabbit, #3045) and anti-LOX-1 (1:200, Santa Cruz Biotechnology, Inc., Dallas, TX, USA). Membranes were further probed with an antibody against β-actin (1:3000 dilution, Mouse, Santa Cruz Biotechnology Inc.), which served as a loading control. Subsequently, they were washed three times with TBS/0.1%Tween20 for 10 min and then incubated with the appropriate horseradish peroxidase (HRP)-conjugated secondary antibodies for 1 h at room temperature (RT) (Anti-rabbit; 1:10,000, Invitrogen, Life Technologies, Paisley, UK, Anti-rabbit; 1:10,000, #BA1054-1, Boster, CA, USA, and anti-mouse; 1:10,000, #BA1050-1, Boster, CA, USA). Each immunoblot analysis was performed in triplicate, and representative blots are shown. The expression of each protein (SIRT1, LC3, p62, ATG5, ATG13, PI3K-CIII, Beclin-1, ULK1, Acetyl-NF-κB p65, LOX-1), normalized relative to the housekeeping protein (β-actin), was quantified with ImageJ software based on band density.

### 2.6. Immunofluorescence

OA chondrocytes after treatment with Resveratrol or normal chondrocytes under serum starvation conditions (~200,000 cells) were grown on coverslips in 6-well plates, and were fixed in ice-cold absolute methanol at −20 °C for 10 min, or in 4% paraformaldehyde (PFA) at RT, for 20 min. Fixed samples were then incubated with anti-LC3 (1:200, Cell Signaling Technology, LC3A/B Rabbit #4108), anti-p62 (1:200, Cell Signaling Technology, SQSTM1/p62 Mouse #88588) and anti-LOX-1 (1:100, Santa Cruz Biotechnology, Inc.) and the appropriate fluorescent dye-conjugated secondary antibodies (1:500, Alexa Fluor 594, Molecular Probes) (1:400 Alexa Fluor 488, Molecular Probes). Coverslips were then embedded in 10 μL of Vectashield mounting medium (Vector Laboratories, CA, USA) containing 4,6-diamidino-2-phenylindole (DAPI) to visualize nuclei and observed on a ZEISS Axio Imager.Z2 fluorescent microscope. Images were analyzed using ImageJ software. For calculations, at least 5 randomly selected fields were analyzed for each condition by two independent observers blinded to the origin of the sample (Normal or OA chondrocytes, treated or not treated (Control)). Each observer counted at least 200 cells for each time point and the means of their counts were used for the statistical analysis.

### 2.7. Immunoprecipitation

Normal or osteoarthritic chondrocytes treated or not with HCQ for 2 h were washed with cold PBS and then were lysed (30 min, 4 °C, vortex every 5 min) in lysis buffer containing 50 mM Tris–Hcl pH 7.4, 150 mM NaCl, 1 mM EDTA, 1% Triton and supplemented with a protease and phosphatase inhibitor cocktail (Thermo Fisher Scientific, Waltham, MA, USA). After centrifugation, the supernatants were collected in 0.5 mL tubes and the sample volume was adjusted to 100 μL with lysis buffer. After this, samples were incubated O/N with 4 μL anti SIRT1 antibody 1:25 (Cell Signaling Technology, SIRT1 Rabbit #2496) on a rotating device at 4 °C. The next day, 20 μL of protein A/G PLUS Agarose beads (sc-2003) were added and incubation continued for 4–6 h at 4 °C on the rotator. After centrifugation (2500 rpm, 5 min, 4 °C), beads were collected and washed 5 times with cold PBS and then proteins bounded to beads were eluted by 40 μL 2× sample reducing buffer, vortexed and then kept for 5 min at 95 °C.

### 2.8. Statistical Analysis

SPSS 25.0 software was used for the statistical analysis of the results. *p*-values less than 0.05 were considered statistically significant, as indicated by the asterisk symbols in the graphs: * = *p* < 0.05; ** = *p* < 0.01; *** = *p* < 0.001. Results are reported as mean ± standard error (means ± S.Ε.). The experimental protocols were performed in triplicate (using at least 3 different healthy donors and 3 different OA patients) unless otherwise stated. Pearson’s correlation coefficient was used to analyze correlative relationships.

## 3. Results

### 3.1. SIRT1 and LOX-1 Protein Expression Are Inversely Correlated in OA Chondrocytes

**Figure 1:**
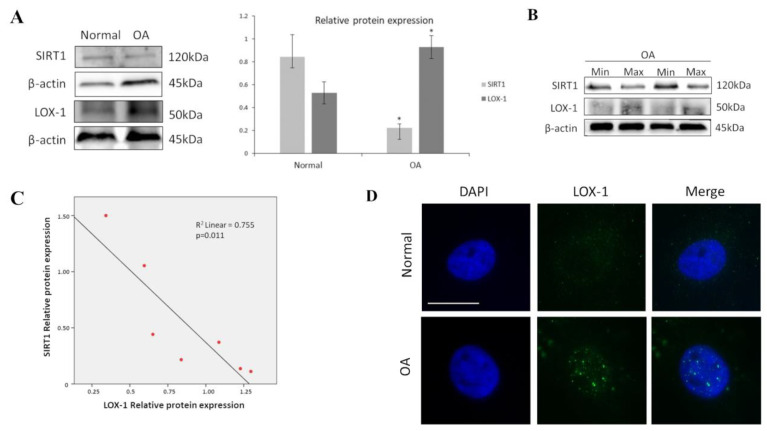
SIRT1 and LOX-1 protein expression are inversely correlated in OA chondrocytes. (**A**) Representative immunoblot showing differential protein expression of SIRT1 and LOX-1 in OA chondrocytes. Antibody against β-actin was used as loading control. *p*-values less than 0.05 were considered statistically significant, as indicated by the asterisk symbols in the graphs: * = *p* < 0.05. (**B**) Differential protein expression of SIRT1 and LOX-1 in OA chondrocytes isolated from joints with maximal damage (max) compared to the same joints with minimum degradation (min). (**C**) Pearson’s rank correlation analysis was used to determine the association between expression levels of SIRT1 and LOX-1 in OA chondrocytes. R^2^ Linear: coefficient of determination (goodness-of-fit measure of the model). (**D**) Immunofluorescence analysis of LOX-1 in OA chondrocytes. Representative images of normal and OA chondrocytes stained with anti-LOX-1 antibody and the appropriate secondary antibody. Nuclei were stained with DAPI (blue). Images were captured with 100× objective lens of the fluorescent microscope used. Scale bar 25 μm.

Since, as mentioned, SIRT1 was found in previous studies to be downregulated in OA chondrocytes and LOX-1 to be aberrantly expressed in OA tissues, we firstly aimed at assessing their protein expression levels in OA and normal chondrocytes. Our results showed that SIRT1 protein levels were significantly reduced in OA compared to normal chondrocytes (*p* < 0.05) (Figure 1A). SIRT1 protein levels were also significantly reduced in OA chondrocytes isolated from cartilage with maximal damage (max) compared with cartilage areas of the same joint with minimum degradation (min) (Figure 1B). We also found that LOX-1 protein expression was significantly upregulated in OA chondrocytes compared to normal and was inversely correlated with SIRT1 expression (*p* < 0.05) (Figure 1C). LOX-1 expression and localization in OA and normal chondrocytes was also assessed by immunofluorescence analysis. In normal chondrocytes, LOX-1 demonstrated a diffuse staining, indicating that LOX-1 was expressed at low levels under physiological conditions. Immunofluorescence analysis of LOX-1 in OA chondrocytes, on the other hand, showed a different, more prominent pattern, appearing in “large dots” with an apparent perinuclear/nuclear concentration (Figure 1D).

### 3.2. SIRT1 Regulates LOX-1 Expression via NF-κB Pathway in OA Chondrocytes

**Figure 2:**
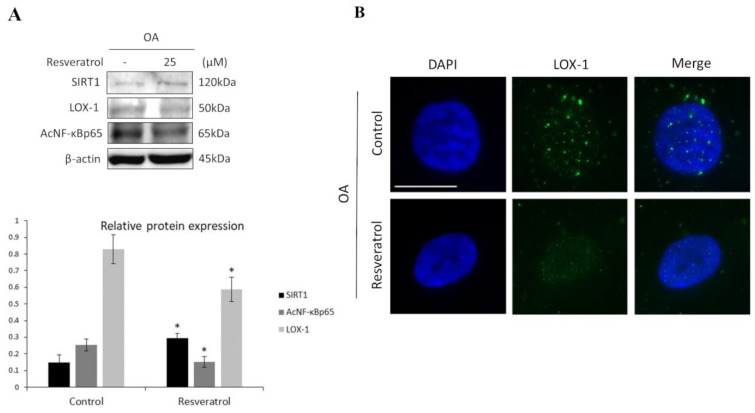
SIRT1 regulates LOX-1 expression via the NF-κB pathway in OA chondrocytes. (**A**) Representative immunoblot showing differential relative protein expression of SIRT1, AcNF-κΒp65 and LOX-1 in OA chondrocytes treated or not with 25 μΜ Resveratrol. Antibody against β-actin was used as loading control. *p*-values less than 0.05 were considered statistically significant, as indicated by the asterisk symbols in the graphs: * = *p* < 0.05. (**B**) Immunofluorescence analysis of LOX-1 in OA chondrocytes treated with 25 μΜ Resveratrol. Representative images of OA chondrocytes stained with anti-LOX-1 antibody and the appropriate secondary antibody. Nuclei were stained with DAPI (blue). Images were captured with 100× objective lens of the fluorescent microscope used. Scale bar 25 μm.

It is known that SIRT1 targets NF-κB for deacetylation. When acetylated, NF-κB binds at the promoter of LOX-1, inducing its transcription [29,30]. To investigate the role of the SIRT1-mediated regulation of LOX-1 expression in OA chondrocytes, we evaluated NF-κB acetylation levels after 72 h treatment with Resveratrol, which is a well-established SIRT1 activator. Western blot results demonstrated SIRT1 upregulation and reduced levels of acetylated NF-κB after Resveratrol treatment, compared to non-treated cells (Control) (*p* < 0.05). In accordance with our initial hypothesis, the analysis also demonstrated reduced LOX-1 protein expression in treated OA chondrocytes, compared to the control condition (*p* < 0.05) (Figure 2A). To further support our findings, microscopic analyses of LOX-1 expression in Resveratrol-treated chondrocytes showed an expression and localization pattern that resembled the LOX-1 pattern previously observed in normal chondrocytes, as compared to control OA chondrocytes in which LOX-1 appeared in dots with perinuclear/nuclear localization (Figure 2B).

### 3.3. Autophagy Induction through SIRT1 in Resveratrol-Treated OA Chondrocytes

**Figure 3:**
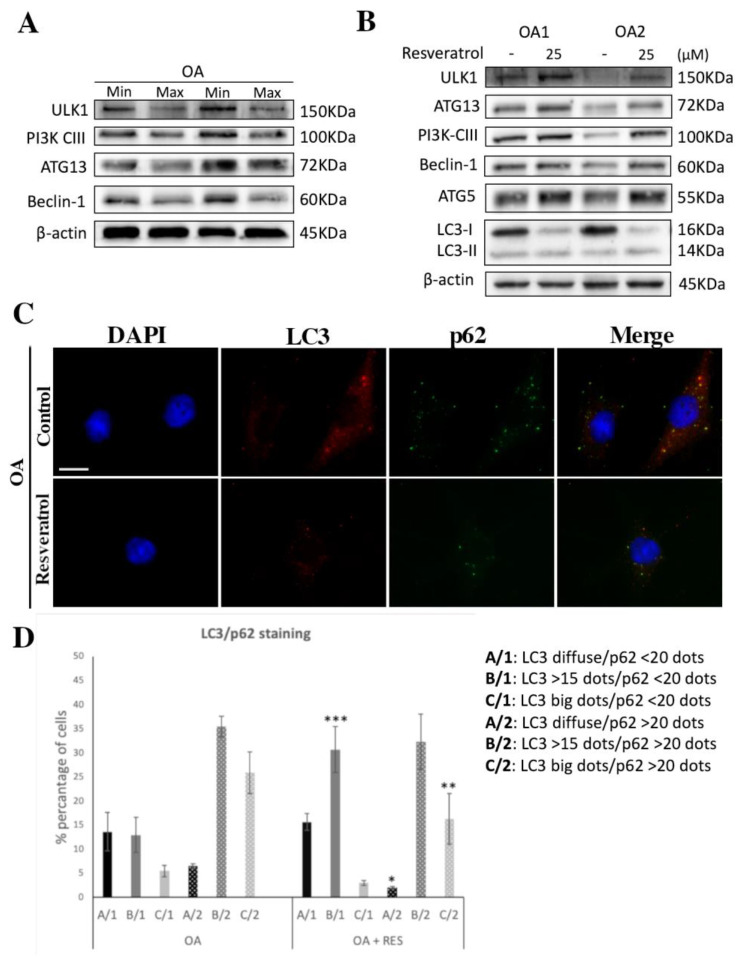
Autophagy induction through SIRT1 in Resveratrol-treated OA chondrocytes. (**A**) Characteristic immunoblot showing protein levels of key molecules of autophagy (ULK1, PI3K CIII, ATG13, Beclin-1) in OA chondrocytes of cartilage regions with minimal (min) and maximal (max) lesions. Antibody against β-actin was used as loading control. (**B**) Representative immunoblot showing protein levels of key molecules of autophagy (ULK1, PI3K CIII, ATG13, Beclin-1, ATG5, LC3I/II) in OA chondrocytes treated or not with 25 μM Resveratrol. Antibody against β-actin was used as loading control. (**C**) Representative images of OA chondrocytes under normal conditions or after 25 μM Resveratrol treatment, stained with anti-LC3 (red)/anti-p62 (green) and the appropriate secondary antibody. Nuclei were stained with DAPI (blue). Images were captured with 40× objective lens of the fluorescent microscope used; scale bar 25 μm. (**D**) Graph showing the percentage of cells with different combinations of LC3/p62 stain in OA chondrocytes treated or not with 25 μM Resveratrol. Values shown are the means ± S.E. * *p* < 0.05, ** *p* < 0.01 and *** *p* < 0.001 vs. Resveratrol-treated OA chondrocytes.

In previous studies from our group and others [11,41,42,43], autophagy was found to be downregulated in OA chondrocytes as compared to normal cells. Here, we show that autophagy-related genes ULK1, ATG13, PIK3-CIII and Beclin-1 are also downregulated in OA chondrocytes derived from cartilage areas with advanced lesions (max) compared to min (Figure 3A). Moreover, we show that Resveratrol treatment of OA chondrocytes resulted in increased protein levels of autophagy-related genes (ULK1, ATG13, PIK3-CIII, Beclin-1 and ATG5) (Figure 3B), indicating the induction of autophagy. The stimulation of autophagy after Resveratrol treatment in OA chondrocytes was further confirmed by the analysis of LC3II/I ratio protein levels and LC3II/I p62 localization patterns, most commonly used markers for autophagolysosome formation. Autophagy induction leads to LC3-I conversion to LC3-II. LC3-II protein expression upregulation seems to correlate with the stimulation/initiation of autophagy [44], and since cells are selectively degraded by autophagy [45], LC3-II expression levels provide an estimate of the autophagic flux. After Resveratrol treatment, OA chondrocytes showed a significant decrease in LC3-I with a parallel increase in LC3II (Figure 3B), indicative of autophagy stimulation. Moreover, LC3/p62 localization was detected by immunofluorescence. For LC3 staining, three different patterns were observed (LC3: diffused staining (A); >15 dots (B); large dots (C)), while for p62 staining, two different patterns were observed (p62: <20 dots (1); >20 dots (2)). Thus, the calculated cells were categorized into six different groups, based on LC3/p62 staining. Immunofluorescence analysis showed a significant increase in cells with more than 15 dots of LC3 and <20 dots of p62, an indication of autophagy stimulation, with a parallel decrease in the number of cells with more prominent/larger dots of LC3 and >20 dots of p62, which usually demonstrate a block in the autophagic flux (Figure 3C,D).

### 3.4. SIRT1-Mediated Regulation of LOX-1 Expression Is Autophagy-Dependent in OA Chondrocytes

**Figure 4:**
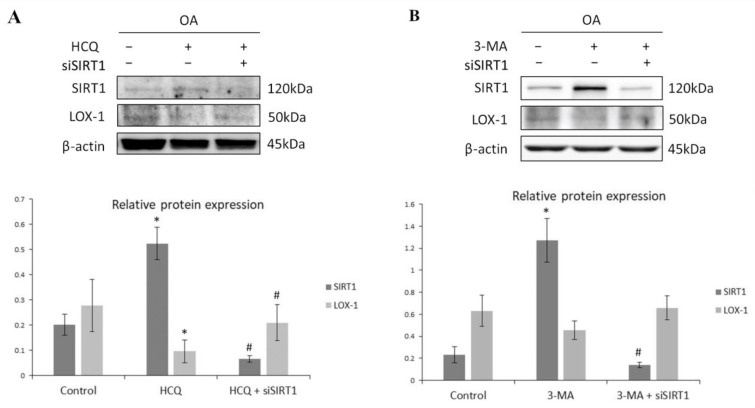
SIRT1-mediated regulation of LOX-1 expression is autophagy-dependent in OA chondrocytes. (**A**) Relative protein expression and quantification of SIRT1 and LOX-1 in OA chondrocytes treated with HCQ and/or siRNA against SIRT1. Antibody against β-actin was used as loading control (* Control vs. HCQ; # HCQ vs. HCQ + siSIRT1). (**B**) Relative protein expression and quantification of SIRT1 and LOX-1 in OA chondrocytes treated with 3-MA and/or siRNA against SIRT1. Antibody against β-actin was used as loading control (* Control vs. 3-MA; # 3-MA vs. 3-MA + siSIRT1). *p*-values less than 0.05 were considered statistically significant, as indicated by the asterisk symbols in the graphs: * = *p* < 0.05.

In order to investigate the possibility that the observed reduced protein levels of SIRT1 in OA chondrocytes were due to deregulation of the autophagic pathway, OA chondrocytes were treated with HCQ and 3-MA, two well-established autophagy inhibitors [46]. Inhibition of the autophagic machinery in the HCQ-treated and 3-MA-treated groups resulted in a significant increase in SIRT1 protein levels (*p* < 0.05), while reducing LOX-1 protein expression, with statistical significance only for the HCQ-treated group (*p* < 0.05), as indicated by Western blot (Figure 4A,B). This effect was reversed in the double-treated groups (HCQ + siSIRT1) and (3-MA + siSIRT1), respectively, where autophagy inhibition was followed by the silencing of SIRT1 expression by a specific siRNA, which prevented any LOX-1 decrease.

**Figure 5:**
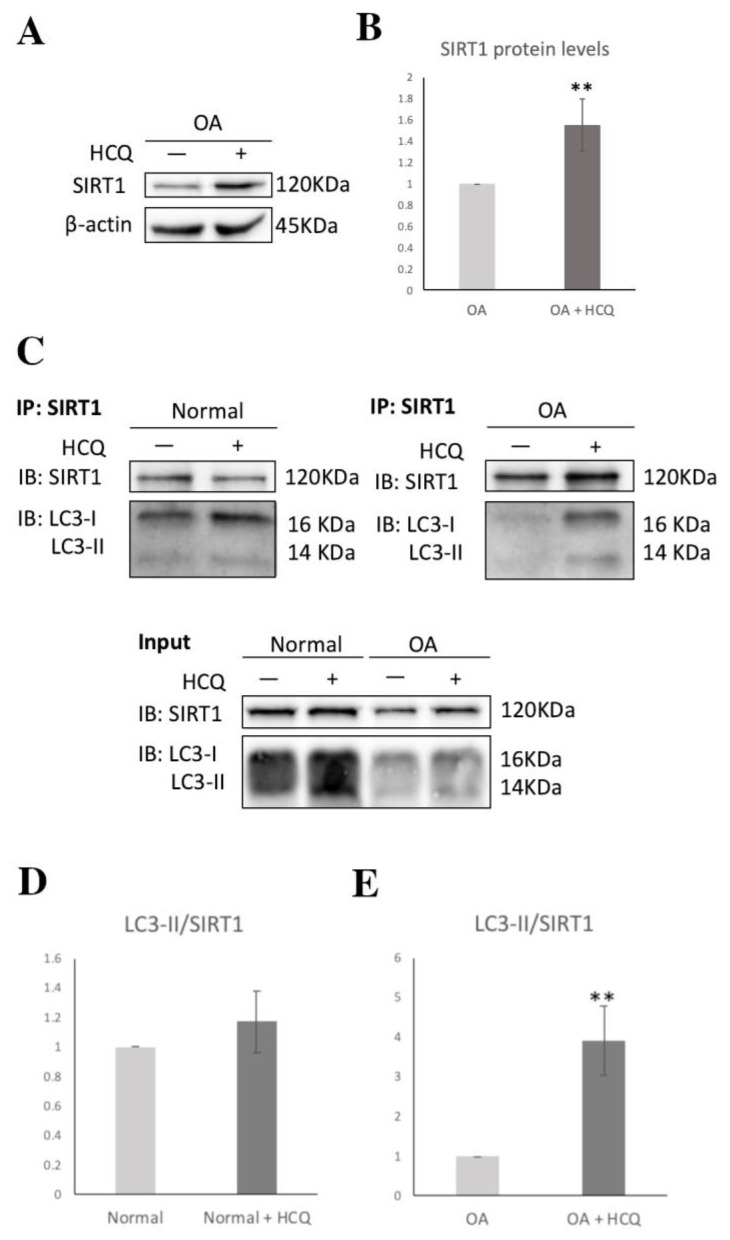
The autophagic degradation of SIRT1 in OA chondrocytes. (**A**) Characteristic immunoblot showing SIRT1 levels under HCQ treatment in OA chondrocytes. Antibody against β-actin was used as loading control. (**B**) Graph demonstrating SIRT1 levels based on band density calculated using ImageJ. Values shown are the means ± S.E. ** *p* < 0.01 vs. the OA under normal conditions. (**C**) IP of extracts from normal and OA chondrocytes treated or not with HCQ. Excessive beads and antibodies were used in IP to capture nearly 100% of SIRT1 protein in the lysates. The Western blot from input is used as indicator of the existence of SIRT1 and LC3-I/II in extracts. (**D**) Graph demonstrating LC3-II IP levels normalized to the SIRT1 IP bands on normal chondrocytes treated or not with HCQ. Band density calculated using ImageJ. (**E**) Graph demonstrating LC3-II IP levels normalized to the SIRT1 IP bands on OA chondrocytes treated or not with HCQ. Band density calculated using ImageJ. Values shown are the means ± S.E. ** *p* < 0.01 vs. the LC3-II/SIRT1 in OA chondrocytes under normal conditions.

To further validate the above hypothesis, we investigated the interaction of SIRT1 with LC3-I/II by performing co-IP in normal and osteoarthritic chondrocytes treated with or without HCQ. The co-IP demonstrated that, in accordance with previous studies [47], SIRT1 interacts with LC3-I/II, both in normal and OA chondrocytes. However, in HCQ-treated osteoarthritic chondrocytes, SIRT1 led to a four-fold increase in the immunoprecipitation of LC3-II (** *p* < 0.01), compared to the untreated group. In contrast, after HCQ treatment in normal chondrocytes, SIRT1 immunoprecipitated LC3-II at similar levels (Figure 5). The above suggests that the increased protein levels of SIRT1 following HCQ treatment are due to the inhibition of the autophagic flux, which further implies that the SIRT1–LC3-II interaction, and therefore SIRT1 selective autophagic degradation, is enhanced in osteoarthritic chondrocytes.

### 3.5. SIRT1 Is Necessary for Autophagy Induction in Articular Chondrocytes

**Figure 6:**
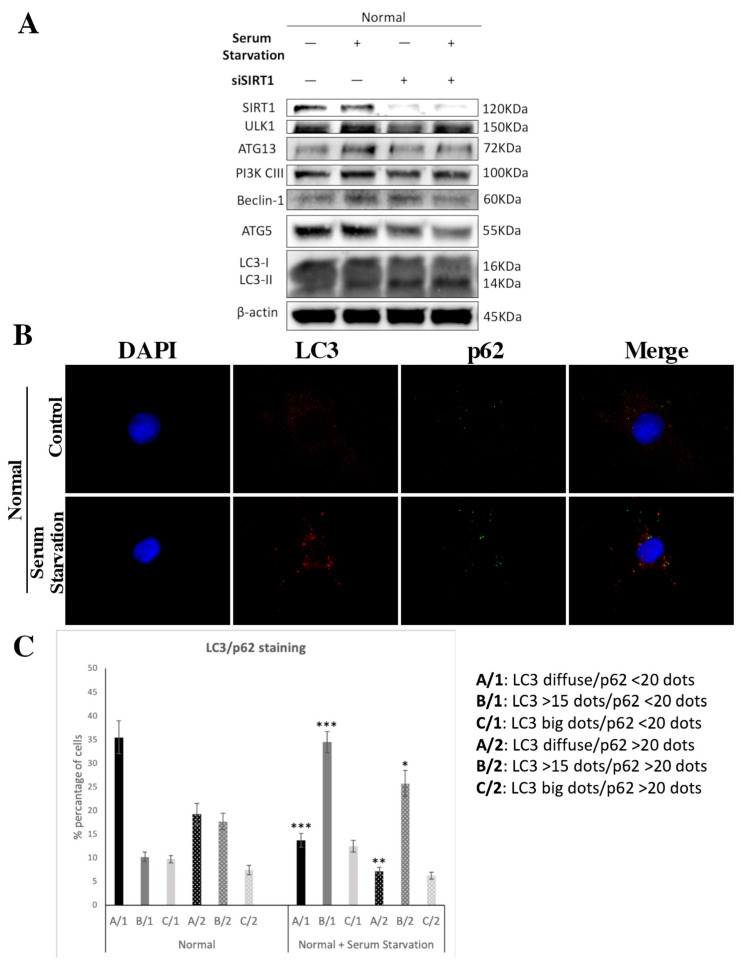
SIRT1 is necessary for autophagy induction in articular chondrocytes. (**A**) Representative immunoblot showing levels of key molecules of autophagy (ULK1, PI3K CIII, ATG13, Beclin-1, ATG5, LC3-I/II) in normal chondrocytes under normal conditions, serum starvation for 3 h, siRNA against SIRT1 and serum starvation after treatment with siRNA against SIRT1. Antibody against β-actin was used as loading control. (**B**) Representative images of normal chondrocytes under normal conditions or after serum starvation, stained with anti-LC3 (red)/anti-p62 (green) and the appropriate secondary antibody. Nuclei were stained with DAPI (blue). Images were captured with 40× objective lens of the fluorescent microscope used; scale bar 25 μm. (**C**) Graph showing the percentage of cells with different combinations of LC3/p62 stain in normal chondrocytes under normal conditions or after serum starvation. Values shown are the means ± S.E. * *p* < 0.05, ** *p* < 0.01 and *** *p* < 0.001 vs. serum-starvation-treated normal chondrocytes.

To further explore the interplay between SIRT1 and autophagy, we investigated the necessity of SIRT1 for the induction and regulation of autophagy in normal chondrocytes. We cultured normal chondrocytes in serum-free medium for 3 h, as serum starvation is a common method to induce autophagy [48]. After serum starvation treatment, normal chondrocytes showed increased protein levels of ULK1, ATG13, PIK3-CIII, Beclin-1 and ATG5, indicating an induction in the autophagic pathway (Figure 6A). LC3-II protein expression upregulation also implies the stimulation/initiation of autophagy. In immunofluorescence analysis for LC3 staining, three different patterns were observed (LC3: diffused staining (A); >15 dots (B); large dots (C)), while for p62 staining, two different patterns were observed (p62: <20 dots (1); >20 dots (2)). The calculated cells were categorized into six different groups, based on LC3/p62 staining. Autophagy induction was observed as a significant increase in cells with more than 15 dots of LC3 (instead of diffuse staining) and increased p62 dots (Figure 6B,C). Importantly, when normal chondrocytes were treated with siRNA against SIRT1 and cultured in serum-free medium, they did not seem to be able to induce autophagy, as was clearly demonstrated by the unchanged protein levels of autophagy-related genes (Figure 6A). The above results indicate the dependance on SIRT1 of articular chondrocytes for autophagy induction.

## 4. Discussion

Ageing, together with metabolic imbalance, is among the key risk factors for OA development [5,6]. SIRT1 has been initially identified as a major nutrient-sensitive regulator and longevity factor in OA and, when downregulated, results in inadequate management of chondrocyte homeostasis [49]. Interestingly, SIRT1 and NAD+ co-factor deficiency is prominent in OA patients and experimental models of skeletal diseases [50,51,52,53,54,55]. LOX-1, on the other hand, has been initially identified as the major ox-LDL receptor in endothelial cells, related to the pathogenesis of atherosclerosis [56]. OA and atherosclerosis share a common pathophysiology, as they are both age-related conditions with a strong metabolic component [57]. LOX-1 overexpression results in aberrant ox-LDL influx in OA articular chondrocytes that affects cell viability by promoting cartilage calcification and ECM hydroxyapatite-calcium modulation [32,58,59].

In the present study, SIRT1 protein levels were found to be significantly downregulated, while LOX-1 protein levels were significantly upregulated in OA chondrocytes compared to normal. Decreased SIRT1 expression levels and LOX-1 overexpression have been previously reported in OA tissues [32,60]. It has, also, been recently reported that SIRT1 expression exhibits an age-related decline, as similar lower levels were detected in aged and OA chondrocytes as compared to chondrocytes from young individuals [38]. We additionally showed that SIRT1 and LOX-1 are inversely correlated in OA chondrocytes. We also demonstrated that SIRT1 expression loss in OA chondrocytes derived from cartilage areas with advanced lesions (max) was inversely proportional to LOX-1 protein expression, while LOX-1 expression was lower in the OA chondrocytes of minimally eroded cartilage (min), where SIRT1 was expressed in higher amounts. The latter further supports the negative correlation among SIRT1/LOX-1 protein expression levels, but the underlying mechanism remains poorly understood.

It is known that NF-κB is a master regulator, mainly of inflammation, and a transcription factor of many central components of a variety of pathways [61,62,63]. NF-κB activation is a complex procedure that involves various interactions with pro-inflammatory cytokines (canonical pathway) and non-death-receptor members of the TNF family (non-canonical), as well as the phosphorylation and ubiquitination of signaling proteins that mediate IKK activation [64,65,66]. Nevertheless, it is also known that NF-κB is subjected to the direct acetylation and deacetylation of the RelA/p65 subunit, leading to NF-κB activation and inactivation, respectively [67]. SIRT1 targets NF-κB for deacetylation in human articular chondrocytes, mitigating its binding affinity to its target genes, thus inhibiting their transcription [68,69]. When acetylated, NF-κB takes part in LOX-1 transcription by binding to its promoter [29,30,70,71]. Resveratrol (3,4′,5-trihydroxystilbene), an active polyphenol found in our food sources and a SIRT1 activator [33], has been recently characterized as a protective flavonoid against the human OA chondrocyte injury induced by IL-1β via the NF-κB signaling pathway [72]. To our knowledge, our results show, for the first time, that in OA chondrocytes, due to SIRT1 downregulation, NF-κB is acetylated, contributing to LOX-1 overexpression. Upon Resveratrol-mediated SIRT1 activation, NF-κB acetylation levels were decreased, followed by LOX-1 downregulation, suggesting that SIRT1 regulates LOX-1 expression via NF-κB deacetylation in OA chondrocytes.

Resveratrol has been shown to have protective properties against age-related diseases, such as diabetes and heart disease, and has been described as a chemopreventive agent against cancer [73]. Moreover, Resveratrol can trigger autophagy in cells from different organisms, extend lifespan and ameliorate the fitness of human cells undergoing metabolic stress [74]. Previous studies have reported that Resveratrol significantly prevents the destruction of OA cartilage by activating SIRT1 and suppressing the expression of HIF-2α and catabolic factors [75]. Recent evidence suggests that SIRT1 induction significantly increases autophagy in aged chondrocytes, by directly deacetylating crucial autophagy proteins such as ATG5, ATG7, Beclin-1 and LC3 in lysine residues, while reduced expression of SIRT1 reduces autophagy in young chondrocytes [47]. However, it remains unclear whether Resveratrol ameliorates OA cartilage destruction by regulating autophagy. To our knowledge, our results demonstrate, for the first time, that upon Resveratrol treatment of OA chondrocytes, SIRT1 is upregulated and the expression of key autophagy markers (ATG5, ATG13, PI3K CIII, Beclin-1 and ULK1) is increased, while the LC3-II/LC3-I ratio shows autophagy induction. SIRT1 and autophagy have been shown to be dysregulated in chondrocytes from aged and OA cartilage, suggesting that a direct functional relationship exists between the two longevity-linked factors [11,47].

In an attempt to address the question of whether SIRT1 is definitely required for autophagy induction, we stimulated autophagy with serum starvation in normal chondrocytes with or without parallel silencing of SIRT1 via siRNA transfection. We found that while serum starvation induces autophagy, parallel downregulation of SIRT1 prevented it, implying the necessity of SIRT1 for autophagy activation in normal chondrocytes.

Autophagic and metabolic homeostasis intertwine in a complicated way, which further demonstrates the complexity of autophagic activity [24]. It is known that intracellular lipids are stored in lipid droplets (LDs). During fasting, the lysosomal degradation of LDs (lipophagy) is crucial [25,26,27] because, in the case of autophagy inactivation, lipids accumulate in cells [76]. Moreover, it has been shown that, in the state of nutrient starvation, autophagosomes and lysosomes are targeted to the LD surface to orchestrate lipophagy [77]. On the contrary, lipids may also affect the activity of autophagy. The increased lipid uptake in β-cells can induce autophagy, while long-term lipid accumulation causes autophagy inhibition [78]. SIRT1 has been reported as a factor that can facilitate both autophagy and lipophagy. Specifically, the necessary and sufficient condition for autophagy/lipophagy induction in hepatic cells is ATGL activation. On the other hand, lipophagy can facilitate the ATGL-mediated LD catabolism and oxidation of hydrolyzed fatty acids (FAs) [79,80]. Moreover, SIRT1 is necessary for the induction of PGC-1α/PPAR-α target genes as part of the oxidative metabolism, in response to increased ATGL-mediated lipolysis [81]. Taken together, these studies indicate that SIRT1 could promote autophagy/lipophagy via ATGL signaling, as a compensative mechanism to control hepatic LD catabolism and FA oxidation.

Interestingly, a duality of the autophagic machinery purpose, especially for OA chondrocytes’ survival, has been recently described, indicating that autophagy is not entirely beneficial [22]. Recent evidence supports the fact that the role of autophagy in OA pathogenesis is binary, as, besides having a protective adaptive response against cytotoxicity in early OA, in late OA, overactivity of autophagy can induce cell death [12,23,82]. In an attempt to highlight the master regulatory role of SIRT1 in both procedures of autophagy and lipid metabolism, we treated OA chondrocytes with HCQ and 3-MA, as autophagy inhibitors. Our results showed that upon both HCQ and 3-MA treatments, SIRT1 protein expression levels were increased, while LOX-1 followed the inverse trend. This outcome was prevented in double-treated OA chondrocytes with HCQ + siSIRT1 and 3-MA + siSIRT1, where autophagy inhibition with parallel SIRT1 silencing resulted in LOX-1 overexpression, demonstrating, for the first time, an autophagy-mediated mechanism for the SIRT1 regulation of LOX-1, suggesting a novel interplay between autophagy and lipid metabolism.

The above results also suggest that autophagy participates in SIRT1 expression regulation, as autophagy inhibition via HCQ and 3-MA increased SIRT1 expression. Our hypothesis was further supported by recent studies reporting that SIRT1 is subjected to cytoplasmic LC3-mediated autophagosome–lysosome degradation in mammalian cells, in senescence and ageing [83,84]. Given the fact that ageing is a principal risk factor of OA, we hypothesized that the aforementioned autophagic machinery could, possibly, be responsible for the decreased expression levels of SIRT1 in OA chondrocytes. To further investigate this indication, we performed immunoprecipitation in the presence or absence of HCQ and demonstrated that in OA articular chondrocytes, SIRT1 constitutes a selective substrate for autophagy, suggesting that the autophagosome-lysosome pathway contributes to the loss of SIRT1 during OA progression.

## 5. Conclusions

In conclusion, the present study sheds light on the key role of SIRT1 in the complex functional interaction between lipid metabolism and autophagy. We provide novel evidence that SIRT1 takes part in LOX-1 expression regulation, by targeting NF-κB for deacetylation. We also show that the gradual age-related loss of SIRT1 protein expression in OA chondrocytes is autophagy-mediated and suggest a novel possible mechanism that functionally connects lipid metabolism and autophagy in late-stage OA. Overall, our data further clarify the cause for reduced SIRT1 expression in OA chondrocytes, and provide a new framework in our understanding of the central role of SIRT1 in the management of autophagic and metabolic homeostasis in OA.
